# Determination of the urinary concentrations of six bisphenols in public servants by online solid-phase extraction-liquid chromatography tandem mass spectrometry

**DOI:** 10.1007/s00216-024-05386-7

**Published:** 2024-06-18

**Authors:** Andrea Estévez-Danta, Rosario Rodil, José Benito Quintana, Rosa Montes

**Affiliations:** Aquatic One Health Research Center (ARCUS) & Department of Analytical Chemistry, Nutrition and Food Chemistry. R. Constantino Candeira S/N, IIAA Building, Universidade de Santiago de Compostela, 15782 Santiago de Compostela, Spain

**Keywords:** Plastic-related additives, Chemical exposure, Human biomonitoring, Hazardous substances

## Abstract

Bisphenols are widely used as monomers and additives in plastic production. Thus, bisphenol A (BPA) and its most prominent substitutes have been detected in many environmental and human samples. This study proposes an online solid-phase extraction analytical methodology coupled to liquid chromatography with tandem mass spectrometry for the determination of six bisphenols (BPA and bisphenols F (BPF), S (BPS), AF (BPAF), B (BPB), and E (BPE)) in urine samples as an efficient and automated methodology. The method was developed and validated for all bisphenols with good recoveries (92–112%) and repeatability (RSD ≤ 10%) despite the variable matrix effects, except BPAF (which would require a dedicated internal standard), achieving method quantification limits in the 0.05–2.2 ng mL^−1^ range. The methodology was subsequently applied to 435 urine samples from a non-occupational exposure population (civil servants for the regional government) from Santiago de Compostela (Galicia, Spain). Only BPA, BPF, and BPS were positively detected; the last two presented higher detection frequencies than BPA. When the urinary concentrations are extrapolated to human intake and compared to the European Food Safety Agency (EFSA) tolerable daily intake (TDI) of 2 × 10^−4^ µg kg^−1^ day^−1^ (TDI), all BPA positively identified samples would surpass this threshold. Although no TDI exists currently for the other two identified bisphenols, it is evident that human exposure to bisphenols should be limited. Finally, the results stratification by gender revealed higher levels of exposure to BPF in the women group.

**Supplementary Information:**

The online version contains supplementary material available at 10.1007/s00216-024-05386-7.

## Introduction

Bisphenols pose unique physical and chemical properties, such as clarity, durability, versatility, corrosion protection, mechanical strength, and thermal stability [1]. Thus, they have been largely used by industry in different applications, many of which are related to plastic production. In particular, bisphenol A (BPA) is the most popular bisphenol, being used as a monomer in polycarbonate plastics (70% of BPA global use) and epoxy resins (nearly 30% of total BPA production) [2].

Bisphenols can migrate from plastics to the environment, where they can be uptaken and bioaccumulated by living organisms [3, 4]. Similarly, human exposure to these compounds can also occur, with ingestion being the most significant route, attributed to migration from plastic packaging into consumable products, primarily food or drinking water [5, 6]. In addition, dermal contact or inhalation could also represent an important source of exposure [7, 8].

From a toxicological point of view, BPA has been classified as an endocrine-disrupting chemical [9]. High levels of exposure to BPA can lead to metabolic diseases, such as type 2 diabetes, hypertension, and elevated cholesterol [10, 11], or interference with the immune and reproductive system by hormonal interactions [12, 13]. In 2006, the European Food Safety Agency (EFSA) conducted the initial risk assessment of BPA, prompting the European Commission to establish concentration limitations, particularly focusing on products intended for infants and food contact materials [14, 15]. More recently, EFSA has decided to implement stricter limits on BPA in food contact materials, reducing the tolerable daily intake (TDI) from 4 µg kg^−1^ day^−1^ to 0.2 ng kg^−1^ day^−1^ effective from April 2023 [16]. Similarly, the US Environmental Protection Agency has set an oral reference dose of 0.05 mg kg^−1^ day^−1^ and formulated an action plan in 2010 to mitigate human exposure to BPA [17, 18]. Such restrictions have caused the emergence of other bisphenols aiming at replacing BPA in manufactured products, such as bisphenol F (BPF), bisphenol S (BPS), bisphenol AF (BPAF), bisphenol B (BPB), or bisphenol E (BPE), among others [19]. These compounds have similar properties to BPA; however, their potentially harmful effects on human health have not been deeply investigated [20–22]. In this context, the EFSA has set a migration rate limit of 0.05 mg kg^−1^ in food for BPS [23], but other BPA alternatives have not yet been investigated. Although in 2020, the German government proposed a restriction on BPA and structurally related analog bisphenols [24], the proposal was withdrawn with the intention to re-submit an updated report to ECHA in the upcoming years.

Once bisphenols enter the human body, a fraction can be absorbed and accumulated in different tissues, while another fraction is eliminated via urine and feces as the original chemical or/and as a metabolite [25–27]. Thus, the measurement of bisphenols and their metabolites in urine represents an appropriate approach to evaluate human exposure to these chemicals by human biomonitoring (HBM) studies [28, 29]. HBM can give a reliable and comprehensive picture that can be used to further understand population exposure to these chemicals, since the population data can be stratified by sex, age, etc. However, the analysis of urine can become very laborious and expensive. In particular, a sample preparation step for purification and concentration based on solid-phase extraction (SPE) is usually required [30] before the chromatographic analysis which can be rather time-consuming. Thus, the integration of automation and online extraction methodologies with chromatographic analysis is a major step forward. In this context, online SPE coupling with liquid chromatography tandem mass spectrometry (LC–MS/MS) is one of the best-suited approaches, as it maximizes sample throughput, while providing an adequate preconcentration and clean-up of the urine samples [31, 32].

Hence, the aim of this study was to develop a high throughput analytical methodology for the determination of six bisphenols (BPA, BPF, BPS, BPAF, BPB, and BPE) in urine samples, and, subsequently, use this method to explore human exposure to them. To this end, an online SPE-LC–MS/MS, after enzymatic deconjugation, method was developed and validated. Then, the method was employed to analyze a total of 435 urine samples from civil servants daily working in the city of Santiago de Compostela (Northwest Spain).

## Materials and methods

### Chemicals and reagents

Table S1 compiles information about the structure of target analytes. Analytical standards of BPA, BPAF, BPB, BPE, BPF, BPS, and creatinine and three deuterated analogues (BPA-d6, BPS-d8, and creatinine-d3) used as surrogate internal standards (ISs) were supplied by Sigma-Aldrich (San Luis, MO, USA). Analytical standards as sodium salts of BPA monosulfate (BPA-S), BPA bissulfate (BPA-DS), BPS monosulfate (BPS-S), BPF monosulfate (BPF-S), BPS β-D-glucuronide (BPS-G), and BPA bis-(β-D-glucuronide) (BPA-DG), and analytical standards of BPF β-D-glucuronide (BPF-G) and BPA β-D-glucuronide (BPA-G) were purchased from Toronto Research Chemicals (TRC, Nort York, ON, Canada).

LC–MS grade methanol (MeOH), LC–MS grade water (ultrapure water), LC–MS grade acetic acid, LC–MS grade formic acid, HCl solution (37%), NaOH (98%), β-glucuronidase (from *Helix pomatia*, type H2), ammonia solution in water (25%), NH_3_ in MeOH (7 N), NH_4_F (98%), NaCl (99.5%), Na_2_SO_4_ (99%), KCl (99%), NH_4_Cl (99.8%), CaCl_2_ (97%), urea (99.5%), and creatinine (98%) were supplied by Sigma-Aldrich. Sodium acetate was obtained from Fluka (Steinheim, Germany). KH_2_PO_4_ (99.5%) was supplied by PanReac (Chicago, IL, USA).

### Urine collection

Urine samples were obtained through a volunteer recruitment campaign carried out in collaboration with the General Directorate for Public Health of the Galicia regional Government – *Xunta de Galicia* (Norwest Spain). The sampling lasted 3 weeks in September 2020, and 435 early morning urine (EMU) samples were collected from anonymized volunteers who work as public servants in the regional government administration in the city of Santiago de Compostela. Informed consent was obtained from all volunteers and stored properly. Ethical consent for the study was obtained from the Research Ethics Committee of the Xunta de Galicia (Code 2019/545). Short surveys were also filled out by each volunteer and the following information was collected: sex (male/female), age (in years), tobacco use (yes/no), and residential environment (urban [> 50,000 inhabitants]/suburban [500–50,000 inhabitants]/rural [< 500 inhabitants]). Table S2 summarizes sociodemographic details of the sampled population and the detailed information of all individual samples (sex, age, etc.) is compiled in the ZENODO repository (10.5281/zenodo.10477935). After collection, urine samples were kept in the freezer at − 25 °C until being processed.

### Sample pretreatment

The total concentration of the bisphenols was measured after deconjugation by incubation at 37 °C with the β-glucuronidase (from *Helix pomatia*, type H2) enzyme [33]. The deconjugation experimental conditions, β-glucuronidase concentration (250–850 units), and incubation time (1.5–4.5 h) were optimized by a design of experiments approach using a central composite design (experimental conditions detailed in Text S1).

Once optimal values were set, each sample was filtered through 0.45 µm PVDF syringe-driven filters (Millex, Merck Millipore). Then, 200 µL of filtered urine samples was adjusted to pH 5 with 80 µL of a sodium acetate buffer (1 mM), spiked with 700 units of β-glucuronidase (112 µL of a 5000 units solution in 0.2% NaCl), 20 ng mL^−1^ of the deuterated internal standards mixture (16 µL of a 1 ng µL^−1^ solution), and 392 µL of ultrapure water (total volume 800 µL). Then, samples were incubated at 37 °C for 5 h. Finally, samples were passed through a β-gone β-glucuronidase removal cartridge from Phenomenex (Torrance, CA, USA) in order to eliminate the enzyme and increase SPE and LC columns lifetime, filtered through 0.22 µm PVDF syringe-driven filters and injected into the online SPE-LC–MS/MS system. Urine samples were also employed to estimate urinary creatinine concentrations (analytical details are given in Text S2).

### Analytical determination

**Fig. 1:**
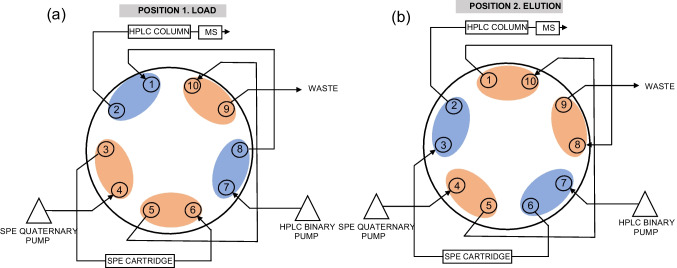
Outline of ten-port switching valve configuration in **a** sample loading and **b** elution and chromatographic separation

The online SPE-LC–MS/MS system involves a sequential process coupling the SPE with the LC–MS/MS through a 10-port valve. This was carried out in an Agilent 1290 Infinity II (Santa Clara, CA, USA) binary solvent pump, for analysis, an Agilent 1260 Infinity II quaternary pump, for online SPE, a thermostatted LC column compartment, a Flexcube module, and a sample manager. MassHunter Data Acquisition and MassHunter Quantitative Analysis software were used for equipment control and data treatment, respectively. The LC system was interfaced to an Agilent 6495 LC/TQ triple quadrupole mass spectrometer. Figure 1 outlines the configuration used.

Each sample or standard was online solid-phase extracted on a Strata-X 25 µm online extraction cartridge (20 × 2.0 mm) from Phenomenex. A dual eluent system consisting of (A1) 15 mM of a sodium acetate buffer at pH 5 in ultrapure water and (B1) MeOH was employed in the quaternary pump. Chromatographic separation was performed on a Luna C18 (150 × 2 mm I.D., 3 µm particle size) column also from Phenomenex with a dual eluent system of (A2) 2 mM of NH_4_F in ultrapure water and (B2) 2 mM of NH_4_F in MeOH, at a flow rate of 0.2 mL min^−1^ (see Table 1 for detailed information on both gradients). Briefly, 500 µL of the deconjugated urine sample, after the pretreatment explained in the previous section, was loaded into the online SPE cartridge in 2 min (0% B1 at 1 mL min^−1^). After a clean-up step with 40% B1, the valve changed to Position 2 (at 7 min) and analytes were eluted towards the chromatographic column with the LC mobile phases. The LC gradient was maintained until 8 min at 2% B2 and then ramped to 100% B2 in 3.5 min, which was maintained for 4 min and finally returned to initial conditions (2% B2), with a total run time of 21 min. Simultaneously, the valve switched back to Position 1 at 15 min and the SPE cartridge was cleaned up with 100% B1 (3.5 min at 1 mL min^−1^) and then equilibrated at 0% B1 for the next injection (see details in Table 1).

**Table 1:**
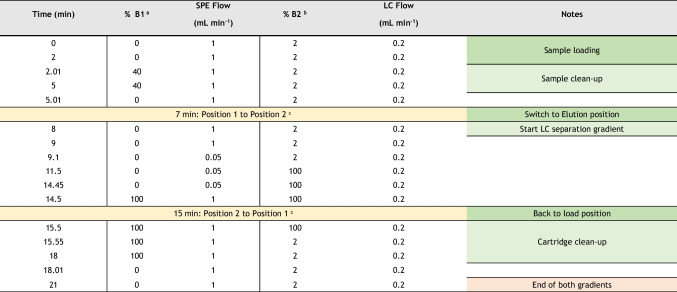
Program of the quaternary (SPE, % B1) and binary (LC, % B2) pumps and switching 10-port valve. ^a^Dual eluent system of the SPE quaternary pump (A1: 15 mM sodium acetate buffer at pH 5 in ultrapure water, and B1: MeOH). ^b^Dual eluent system of LC binary pump (A2: 2 mM of NH_4_F in ultrapure water and B2: 2 mM of NH_4_F in MeOH). ^c^See Fig. 1

A Jet Stream electrospray ionization (ESI) source operating in negative mode was the interface between the LC system and the 6495 LC/TQ triple quadrupole mass spectrometer at a fixed capillary and nozzle voltage of 2.5 and 0.5 kV, respectively. The iFunnel voltage was set at 60 V/70 V (high/low-pressure RF). Nitrogen was used as sheath gas at 12 L min^−1^ and 400 °C and also as desolvation gas at 14 L min^−1^ and 120 °C. Analyses were performed by MS/MS in selected reaction monitoring (SRM) mode using 166 V as fragmentor voltage. Table 2 summarizes the retention times (RT), SRM transitions (*Q*_n_), optimal collision energies (CE) for the target bisphenols, and internal standards. Three SRM transitions were recorded for each analyte, except for BPB and BPE for which only two transitions were obtained, and ion ratios were monitored in order to ensure proper identification of analytes [34]. Only one transition was recorded in the case of ISs.

**Table 2:** Chemical formulae, retention time (RT), transitions (*Q*_n_) used for quantification (*Q*_1_), and confirmation (*Q*_2_ and *Q*_3_), ratio between the transitions, optimal collision energy (CE) values, and compounds used as internal standards (IS)

Compound	Chemical formulae	RT (min)	*Q*_1_ (*m/z*)	CE (eV)	*Q*_2_ (*m/z*)	CE (eV)	*Q*_2_/*Q*_1_	*Q*_3_ (*m/z*)	CE (eV)	*Q*_3_/*Q*_1_	IS
BPA	C_15_ H_16_ O_2_	13.8	227 > 211	37	227 > 133	25	0.87	227 > 93	57	0.23	BPA-d6
BPAF	C_15_ H_10_ F_6_ O_2_	14.0	335 > 265	21	335 > 197	45	0.079	335 > 315	21	0.031	BPA-d6
BPB	C_16_ H_18_ O_2_	13.9	241 > 212	17	241 > 226	17	0.088	-	-	-	BPA-d6
BPE	C_14_ H_14_ O_2_	13.7	213 > 197	33	213 > 198	17	0.41	-	-	-	BPA-d6
BPF	C_13_ H_12_ O_2_	13.6	199 > 93	21	199 > 105	21	0.59	199 > 77	29	0.49	BPA-d6
BPS	C_12_ H_10_ O_4_ S	13.3	249 > 108	29	249 > 156	21	0.37	249 > 92	37	0.72	BPS-d8
BPA-d6	C_15_ H_10_^2^H_6_ O_2_	13.8	233 > 138	25	-	-	-	-	-	-	-
BPS-d8	C_12_ H_2_^2^H_8_ O_4_ S	13.4	257 > 112	29	-	-	-	-	-	-	-

### Method performance

BPA and BPS were quantified using their surrogate analogues as IS. BPA-d6 was also used as IS for the remaining bisphenols (Table 2) as it provided better performance when compared to BPS-d8.

Calibration curves for the final method were prepared in ultrapure water and ranged from the MQL to 50 ng mL^−1^, IS level set to 20 ng mL^−1^. The lowest concentration of the calibration curve was selected as a function of the MQL for each analyte. Originally 11 standards were prepared in order to have a calibration curve with 11 points (0.1, 0.25, 0.5, 0.75, 1, 2.5, 5, 7.5, 10, 25, 50 ng mL^−1^ + 20 ng mL^−1^ of IS in all cases); however, this was only used for BPS. For the other compounds, the lowest level was selected depending on the respective MQL; thus, BPA and BPF calibration curves consisted of 6 points (2.5–50 ng mL^−1^) and BPB, BPE, and BPAF calibration curves consisted of 7 points (1–50 ng mL^−1^). Online SPE-LC–MS/MS method performance was investigated in terms of linearity, trueness, precision, method detection limits (MDL), and method quantification limits (MQL) (Table 3). Moreover, the slope and intercept estimated and their standard errors are included in Table S3. Trueness and precision (*n* = 5) were assessed by recovery studies performed in ultrapure water at 10 ng mL^−1^ and in (both deconjugated and non-deconjugated) urine at three levels, 5, 50, and 200 ng mL^−1^ of all analytes (20 ng mL^−1^ of ISs). Urine aliquots spiked only with IS (non-spiked) were processed at the same time to account for analyte background levels in this matrix.

**Table 3:** Storage stability and method validation parameters: linearity, trueness and precision, and method detection and quantification limits (MDL and MQL)

Compound	Determination coefficient (*R*^2^)^a^	Trueness and precision %R (%RSD)^b^							MDL (ng mL^−1^)	MQL (ng mL^−1^)	4-week stability %Recovery (%RSD)^c^
		Ultrapure water (10 ng mL^−1^)	Urine 1 (5 ng mL^−1^)		Urine 2 (50 ng mL^−1^)		Urine 3 (200 ng mL^−1^)				
			No deconj	Deconj	No deconj	Deconj	No deconj	Deconj			
BPA	0.9968	103 (4)	106 (7)	103 (9)	86 (3)	89 (3)	107 (7)	107 (9)	0.34	1.1	119 (2)
BPAF	0.9954	87 (4)	79 (3)	11 (9)	57 (15)	30 (6)	67 (8)	40 (7)	0.23	0.76	87 (3)
BPB	0.9977	95 (3)	114 (5)	7 (9)	88 (5)	92 (7)	119 (7)	92 (8)	0.22	0.73	101 (5)
BPE	0.9966	101 (2)	95 (3)	93 (10)	87 (2)	92 (4)	110 (8)	111 (4)	0.18	0.61	98 (1)
BPF	0.9950	104 (5)	87 (8)	120 (9)	103 (5)	114 (4)	109 (7)	112 (6)	0.66	2.2	97 (3)
BPS	0.9965	99 (2)	112 (3)	110 (7)	79 (2)	87(2)	104 (5)	104 (6)	0.015	0.049	102 (3)

^a^Standards for calibration curves were originally prepared with the following concentration (11 points): 0.1, 0.25, 0.5, 0.75, 1, 2.5, 5, 7.5, 10, 25, 50 ng mL^−1^ + 20 ng mL^−1^ of IS. BPS calibration curve consisted of 11 points, BPA and BPF calibration curve consisted of 6 points (2.5–50 ng mL^−1^), and BPB, BPE, and BPAF calibration curve consisted of 7 points (1–50 ng mL^−1^)

^b^IS-corrected recovery (%R) from the nominal spiking value and %RSD from the average measured concentration (*n* = 5), 0.5, 5, and 50 ng mL^−1^ of analytes + 20 ng mL^−1^ of IS (urine, *n* = 5). No deconj = urine samples without deconjugation, deconj = urine samples after enzymatic deconjugation

^**c**^IS-corrected recovery (%R) from the nominal spiking value and %RSD from the average measured concentration. Experiments performed: three urine samples from different donors spiked with 400 ng mL^−1^ of analytes + 20 ng mL^−1^ of IS

Matrix effects (ME) were evaluated as the non-IS corrected recovery of spiked pooled urine samples from 20 different individuals (50 ng mL^−1^). MDLs and MQLs were calculated from the urine samples spiked with 5 ng mL^−1^ and extrapolating this concentration to that corresponding to a signal-to-noise ratio (S/N) of 3 and 10, respectively.

### Bisphenol stability in urine

Bisphenol stability was tested in order to verify if the target compounds were stable in urine samples during the period of sampling and storage. Experiments were performed in triplicate where 1.68 mL of urine was spiked with 320 µL of a mixture solution (final concentration 400 ng mL^−1^), containing the six bisphenols. Then, urine samples were stored in amber glass vials at 22 ± 2 °C. Aliquots of 200 µL (*n* = 3) were collected at time 0 and after 1.5, 3, 5, 8, 24, and 48 h. They were filtered through 0.22 µm hydrophilic PVDF syringe-driven filters, diluted four times with ultrapure water, and spiked with 20 ng mL^−1^ of ISs. Subsequently, these samples were submitted to the final online SPE-LC–MS/MS methodology.

Furthermore, freezing storage stability (− 18 ± 2 °C) was also tested. Three different urine samples, spiked with 400 ng mL^−1^ of analytes, were stored in the same material containers, and kept in the same freezer as volunteers’ urine samples for 4 weeks. On the other hand, creatinine stability has already been demonstrated in previous works, showing good stability for months at different temperatures [35, 36].

### Assessment of human exposure to bisphenols

The concentration of each target compound found in urine samples (in ng mL^−1^) was calculated by the IS calibration method (IS level: 20 ng mL^−1^). Then, creatinine-adjusted concentrations (in µg of bisphenol per g of creatinine) were calculated.

Additionally, human intake of bisphenols (µg kg^−1^ day^−1^) was estimated by considering the average daily urine volume of 1.57 L per individual [37] and the weighted mean of the population weight according to a summary of scientific studies evaluated and published by the NCD Risk Factor Collaboration. The estimated average body mass for men and women in Spain was 84.0 and 65.9 kg, respectively [38].

### Quality control/quality assurance

Quality control (QC) samples were prepared spiking a control pooled urine sample with 100 ng mL^−1^ of analytes (20 ng mL^−1^ of ISs) and processed following the optimized methodology. Two QC samples per batch (each 20 urine samples) were injected to test for method stability (tolerance set to 60–140% range, see “Method performance”). Similarly, different blanks were analyzed within batches to ensure the lack of (cross)contamination. These included system blanks (chromatographic runs without injection), process blanks (ultrapure water spiked with the IS), and storage blanks (sample tubes containing synthetic urine, prepared as described by Laube et al. [39], and stored for up to 4 weeks).

### Statistical analysis

Statistical analysis was performed using the software Statgraphics Centurion 18. Statistical correlation (*α* = 0.05) within the urinary concentration of BPF, BPS, and volunteers’ age was assessed by a Spearman rank correlation (data non-normal distributed). This same software was also used for creating and analyzing the design of experiments used during enzymatic deconjugation optimization (Text S2).

Statistical differences (*α* = 0.05) in the BPF and BPS urinary concentrations between groups of different genders and tobacco use were evaluated by the Mann–Whitney test and statistical differences between the residence environment were evaluated by a Kruskal–Wallis test, with a post hoc Bonferroni correction. Data was graphically presented with the R ggstatsplot package [40]. The values of BPF and BPS below the MDL and MQL were substituted by MQL/2 and MDL/2, respectively. No statistical analysis for BPA was performed due to the high percentage of samples below MQL.

## Results and discussion

### Enzymatic hydrolysis

The hydrolysis of urinary conjugates is critical to estimate the real exposure of bisphenols. β-glucuronidase is the most commonly used enzyme for bisphenols deconjugation at 37 °C [41, 42]. Many commercially available β-glucuronidase enzymes also have sulfatase activity, so that both phase II type of metabolites, glucuronides and sulfates, can be deconjugates. Although some former studies exist, they use different enzymes [30, 42–44]; thus, the deconjugation process was carefully optimized again by a design of experiments approach, as described in Text S1, with the glucuronide and sulfate metabolites available in the laboratory.

**Fig. 2:**
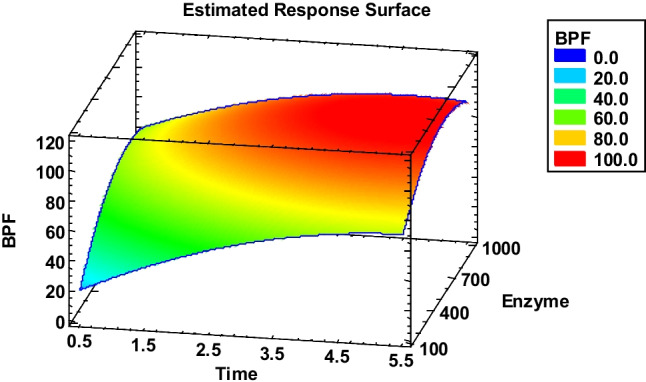
Estimated response surface for BPF, obtained during enzymatic deconjugation DOE optimization

The transformation yield was calculated by measuring the concentration of BPA, BPS, and BPF formed and employed to select the best conditions for the hydrolysis of urinary metabolites. The analysis of the results obtained showed that the formation of BPA (from its mono- and bis-sulfate and mono- and bis-glucuronide) was non-statistically affected by any of the factors (time and enzyme concentration) considered (Figure S1). Thus, BPA conjugates are easily converted to BPA under all conditions tested. On the other hand, the formation of BPF and BPS was statistically affected by both the incubation time and the concentration of enzyme, without significant interaction between factors (Figure S1). As shown in the Pareto charts (Figure S1); however, the deconjugation yield for the metabolites of these two bisphenols is linearly and positively affected by time, while there is a significant curvature on the enzyme concentration (both B and BB terms are statistically significant). As shown in Fig. 2 and S2, where the estimated response surfaces for BPF and BPS are presented respectively, the deconjugation yield does not further increase beyond ca. 600 units of the enzyme. Thus, the optimal values were set at 5 h of incubation and 700 units of β-glucuronidase.

### Chromatographic eluent modifier

Chromatographic separation was performed on a Luna C18 column (150 × 2 mm I.D., 3 µm particle size) at 0.2 mL min^−1^, so as not to surpass the maximum pressure of Strata-X cartridges (260 bar). Ammonium acetate was initially tested, but it afforded much less signal than NH_4_F, which has already been shown in the literature to provide better ionization efficiency in negative mode ESI [45]. Then, different concentrations of NH_4_F were investigated (0.1, 0.2, 0.5, 1, 2, and 5 mM). Finally, 2 mM NH_4_F was selected due to the higher relative response observed in spiked (10 ng mL^−1^, *n* = 3) urine samples (Figure S3). A good peak shape was obtained for the six bisphenols in these conditions (Figure S4).

### SPE optimization

Sample preconcentration and clean-up were performed by online SPE in order to maximize sample throughput. This approach had been already previously employed to determine some of the bisphenols, but not all, considered in this work (Table S4) [42–44, 46, 47]. Among the different available sorbents, Strata-X was used due to its hydrophobic-hydrophilic balance, as this is a co-polymer of styrene–divinylbenzene modified by introducing pyrrolidone groups [48].

A dual eluent system consisting of (A1) ultrapure water and (B1) MeOH at 1 mL min^−1^ was used as a sample carrier and for cartridge clean-up and back-conditioning. Different modifiers of the aqueous phase were considered, viz. 0.1% formic acid, 0.1% acetic acid, and a sodium acetate buffer at pH 5 (in this case at three concentration levels, 5, 15, and 25 mM). The use of 15 mM of sodium acetate buffer at pH 5 was finally selected, as it showed the best peak shape and signal intensity for target analytes, being BPS the most critical compound (Figure S5).

The SPE gradient started at 0% B1 for 2 min to load the sample into the Strata-X cartridge, and after that, a clean-up step of 3 min with different percentages of B1 (10, 20, 40, and 60%) was investigated. Finally, 40% B1 was selected, as the higher % of MeOH allowed the removal of potential matrix interferences without eluting the target compounds from the online SPE sorbent (data not shown). Finally, to improve peak focusing, the quaternary pump was programmed again to aqueous conditions (0% B1) 2 min before the 10-port vale was switched for elution towards the chromatographic column. During the chromatographic gradient, the quaternary pump flow was lowered to 0.05 mL min^−1^ to reduce solvent expenditure, and subsequently, a washing step with 100% B1 of the Strata-X cartridge was included before the subsequent injection (see Table 1 for detailed information).

### Stability of bisphenols in urine

Stability tests were conducted for the target bisphenols in urine following the protocol explained in “Materials and methods.”

The study of sampling stability showed that the six bisphenols were stable for 48 h at room temperature (Figure S6). As urine samples were collected every morning by sanitary employers and stored at 4 °C until they were taken to the laboratory the same morning, degradation during the urine sampling is thus not expected.

The 4-week stability study at − 18 °C did not show any potential loss of bisphenols in urine tubes during these weeks, with % absolute recoveries (%R) between 87 and 119% (Table 3). Some formerly published studies also reported that BPA, BPF, and BPS are chemically stable in frozen urine for several months [42, 49, 50], but the stability of the remaining bisphenols was not tested in the literature before.

### Method performance

Trueness and precision of the online SPE-LC–MS/MS method were assessed through recovery studies performed with ultrapure water and urine (with and without enzymatic deconjugation) at three different concentration levels with three different urine samples (Table 3). For BPA, BPB, BPE, BPF, and BPS, the recoveries ranged between 74 and 120%, with RSDs between 2 and 10%. The exception was BPAF, whose recoveries (even when corrected with BPA-d6) were poor and highly variable, particularly in the deconjugated samples (11 to 40%). MDLs ranged from 0.015 to 0.66 ng mL^−1^, and MQLs from 0.049 to 2.2 ng mL^−1^ (Table 3).

**Fig. 3:**
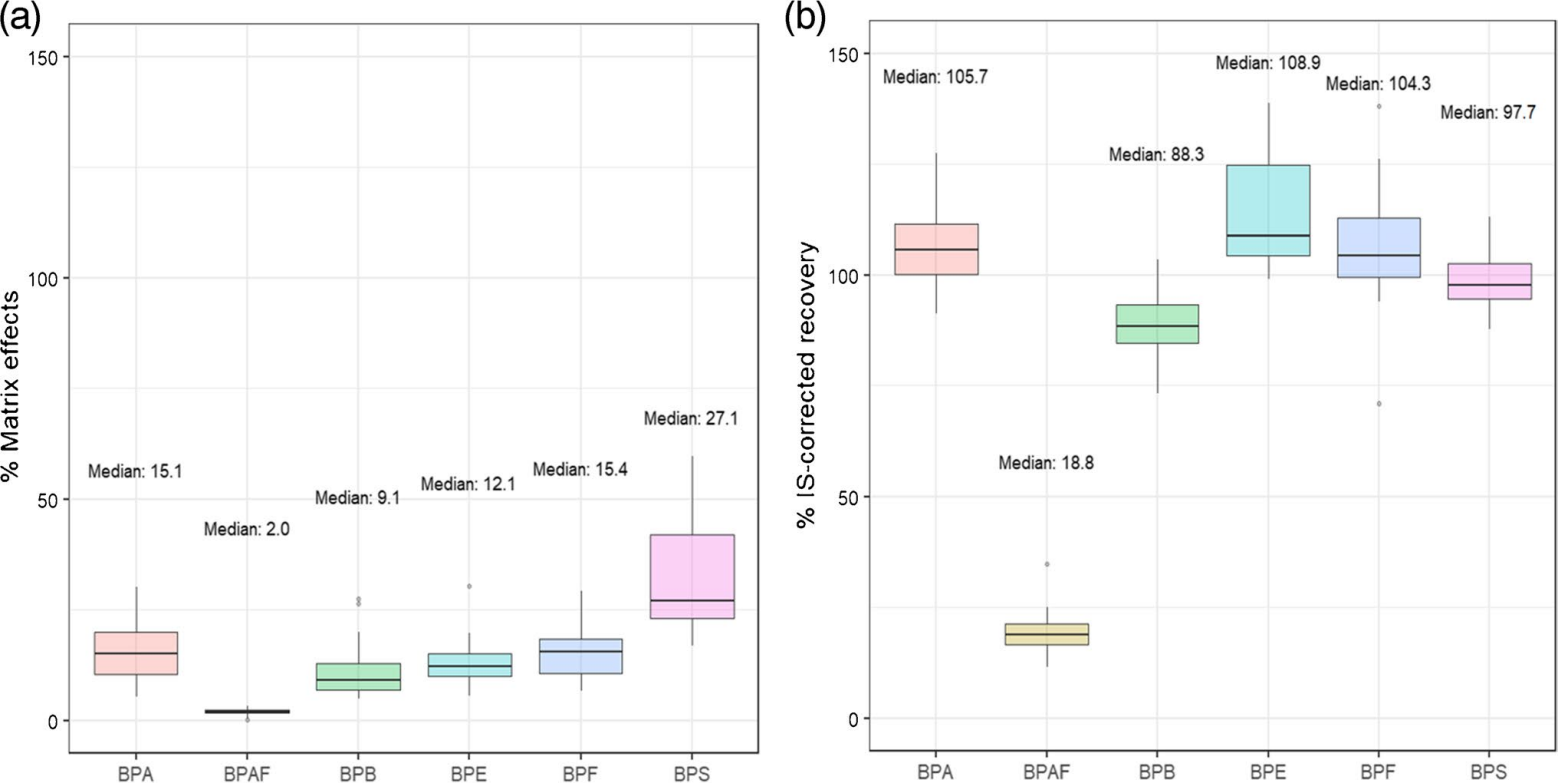
**a** Matrix effects and **b** internal standard corrected recoveries obtained from the analysis of 20 different spiked urine samples

ME were investigated by assessing the absolute recovery with twenty different urine deconjugated samples from different individuals. As presented in Fig. 3a, a strong to very strong signal suppression was observed. Furthermore, such ME were highly variable among samples. For instance, in the case of BPS, % ME average was 32% (median = 27%) but it spanned from 17 up to 60%, depending on the sample. In this case as well as for BPA, the deuterated IS could correct for this variation. Similarly, BPF, BPE, and BPB pose ME values similar to those of BPA, so their response could be corrected by BPA-d6 (Fig. 3b). However, BPAF suffered from an extremely strong signal suppression (average and median ME = 2%), so neither BPA-d6 nor BPS-d8 were suitable ISs to correct its matrix effect (Fig. 3b).

The proposed method was compared to other available online SPE-LC–MS/MS methods for the determination of bisphenols in urine [42–44, 47, 51] (Table S4). Most of the literature studies focus on BPA and the most prominent substitutes, BPF and BPS, while BPAF, BPB, and BPE have been barely studied in urine samples. The trueness for BPA, BPB, BPE, BPF, and BPS was comparable with published methodologies [42, 47]. In the case of BPAF, the only study available [43] used ^13^C_12_-BPA to correct the signal of BPAF, even when reporting very different ME for both compounds (%ME 3% BPA and 69% BPAF) and surprisingly a good IS-corrected recovery in urine was obtained. Nevertheless, in our study, none of the two available deuterated bisphenols was able to provide acceptable trueness for this compound.

As a drawback, for BPAF, it would be necessary to purchase the isotopically labelled analogue or to measure its concentration by standard addition, whenever found in the samples. However, this compound was not detected in the urine samples (see next section).

### Analysis of urine samples

#### Urinary concentrations

The validated methodology was applied to the analysis of a total of 435 samples of human urine (data summarized in Table 4, full data presented in the ZENODO repository: 10.5281/zenodo.10477935). Each analytical batch included two QC samples (100 ng mL^−1^ of analytes) to test for method performance during analysis. Average recoveries in the QC samples remained in the 89–114% for all bisphenols, except, as mentioned for BPAF (19%) and within the 60–140% tolerance range, with good repeatability (%RSD < 5%).

**Table 4:** Overview of urinary levels and estimated human intake of BPA, BPS, and BPF detected in the samples (full data available in ZENODO repository: https://doi.org/10.5281/zenodo.10477935). Statistical parameters calculated by substituting the samples < MQL by MQL/2 and those < MDL by MDL/2

	Concentration (ng mL^−1^)			Creatinine-adjusted concentration (µg g^−1^ creatinine)			Human intake (µg kg^−1^ day^−1^)		
	BPA	BPF	BPS	BPA	BPF	BPS	BPA	BPF	BPS
	n=72^b^	n=421^b^	n=364^b^						
Arithmetic mean ± SD	-^a^	19.6 ± 18.0	2.03 ± 5.35	-^a^	23.1 ± 19.1	2.5 ± 9.2	-^a^	0.47 ± 0.44	0.049 ± 0.129
Geometric mean ± GSD	-^a^	12.4 ± 3.1	0.50 ± 8.66	-^a^	16.6 ± 2.6	0.67 ± 6.64	-^a^	0.30 ± 3.14	0.012 ± 8.657
Median ± SD	< MDL	14.9 ± 18.0	0.90 ± 5.35	< MDL	18.9 ± 19.1	0.99 ± 9.23	< MDL	0.36 ± 0.44	0.022 ± 0.129
Max	103	125	80.1	289	172	173	1.92	3.02	1.93
25% percentile	< MDL	7.89	0.28	< MDL	12.29	0.38	< MDL	0.19	0.007
75% percentile	< MDL	25.4	2.01	< MDL	28.64	2.05	< MDL	0.61	0.049

^a^Not calculated given the high % of samples < MDL

^**b**^Urine samples with concentration level above MQL

Only BPA, BPF, and BPS were detected in urine samples. BPF was the substance more frequently detected (present in 96.8% of the urine samples) with median and maximum concentrations of 14.9 and 125 ng mL^−1^, respectively (median = 18.9 µg g^−1^ and maximum = 172 µg g^−1^ when normalized to creatinine). The second most prevalent bisphenol was BPS (detected in 83.6% of the samples) with median and maximum concentrations of 0.90 and 80.1 ng mL^−1^, respectively (median = 0.99 µg g^−1^, maximum = 173 µg g^−1^ when normalized to creatinine). Finally, BPA was detected in 16.6% of the samples only with a maximum concentration of 103 ng mL^−1^ (289 µg g^−1^ creatinine-corrected). In this case, the median would be < MDL.

According to the literature (see Table S5 for a compilation of recent studies), the HBM study by Frederisken et al. [52] conducted between 2009 and 2017 in young Danish men has identified a slightly decreasing trend in BPA detection frequency, yet a higher increasing trend of substitutes in 2017, especially BPS. Nevertheless, recent studies (Table S5) conducted in Spain [53, 54] and other countries, e.g., Australia and Norway [43, 55], have reported a much higher detection frequency of BPA, with concentrations typically higher than those found in our study, though variable. The literature presents different findings regarding the detection frequency and levels of BPF and BPS so there is a similar prevalence for both alternatives, as in our study. Overall, the results for the population considered in this work point towards a relevant decrease in BPA exposure and an increase in BPF and BPS.

#### Estimated human intake

Urinary concentrations were converted to estimate human intake, as described in “Materials and methods” and compared with the TDI recently set by EFSA at 0.2 ng kg^−1^ day^−1^ for BPA (Table 4). In the case of BPA, all the samples in which this bisphenol was detected (estimated intake up to 1920 ng kg^−1^ day^−1^) would be above the TDI level. Moreover, following the BfR recommendation [24], if the estimated human intake for BPF is compared to TDI for BPA, all samples where BPF was detected (i.e., 421) would again be above that threshold. This fact evidences the need for actions to limit exposure to bisphenols, particularly taking into consideration the uncertain situation as regards the safety of bisphenols other than BPA in terms of their toxicological effects.

#### Influence of sociodemographic factors

Population’s characteristics as well as the results in terms of concentrations and estimated human intake are summarized in Table S2 and Table 4 and detailed in the ZENODO repository (10.5281/zenodo.10477935). The studied population consisted of 67.6% females and 32.4% males in the 32–66 years old range. Also, the population was classified according to tobacco use and residence location (urban, suburban, and rural). The potential association between BPS and BPF and the sociodemographic factors were studied as detailed in the section “Statistical analysis.” BPA was not considered ought to its low detection frequency.

A Spearman’s rank correlation analysis was carried out to investigate the relationship between age and BPF and BPS creatinine-adjusted concentration. There was a significant positive correlation (rho = 0.3194, *p*-value < 0.0001) between BPF and BPS concentrations (Table 5). This fact was previously observed in other studies [44, 52, 55] and may simply be related to the fact that people with higher levels of BPF and BPS are those who are more exposed to plastics and their additives. Furthermore, a very weak positive correlation (rho = 0.09536, *p*-value = 0.0478) was denoted between BPS concentration with age, which may point to the accumulation of this substance by older individuals (Table 5). However, this needs to be taken with caution, given the weak correlation.

**Table 5:** Spearman’s rank correlations coefficient (rho) and *p*-values for BPF, BPS, and participants’ age

		BPS	BPF	Age
BPS	rho		0.3194	0.0953
	*p*-value		** < 0.0001**	**0.0478**
BPF	rho	0.3194		− 0.0852
	*p*-value	** < 0.0001**		0.0768
Age	rho	0.0953	− 0.0852	
	*p*-value	**0.0478**	0.0768	

**Fig. 4:**
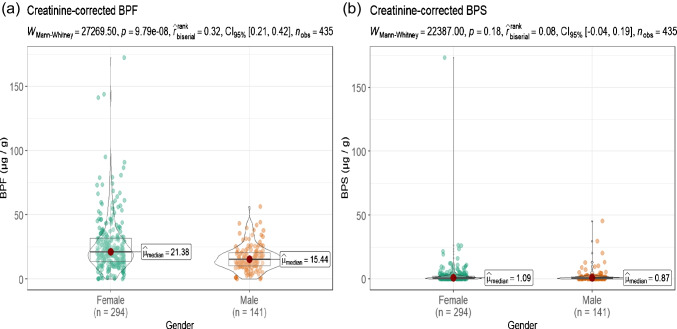
Comparison of creatinine-corrected concentrations (µg g^−1^) according to gender for **a** BPF and **b** BPS

Gender and tobacco use differences in urinary creatine-corrected BPF and BPS concentrations were examined by a Mann–Whitney non-parametric test and residence environment by a Kruskal–Wallis non-parametric test, as detailed in the section of statistical analysis. Higher median concentrations were obtained for females (Fig. 4), but this was only statistically significant for BPF (*p*-value: 9.79 × 10^−8^). Several studies have reported the same phenomenon for bisphenols, where the urine concentration on a ng mL^−1^ basis was higher in men; however, after correction for creatinine, the conclusion was reversed [44, 47, 49]. Hence, when assessing gender-related variations in exogenous substances, it is crucial to consider factors like bioaccumulation or metabolism, which can vary between women and men [56].

The two remaining factors were not statistically significant (Figures S7 and S8). The median creatine-corrected concentration for both bisphenols (Figure S8b) was higher in urban (median 1.09 µg g^−1^ for BPS and 18.92 µg g^−1^ for BPF) and suburban environments median (0.98 µg g^−1^ and 19.32 µg g^−1^ for BPF) than in rural ones (0.72 µg g^−1^ and 16.46 µg g^−1^ for BPF).

## Conclusions

An online SPE-LC–MS/MS methodology has been developed for the determination of six bisphenols (BPA, BPAF, BPB, BPE, BPF, and BPS) and employed for the analysis of 435 human urine samples from a non-occupational exposure population. The method showed satisfactory performance figures, despite the variable matrix effects, for five bisphenols. However, the exception was BPAF, for which the technique exhibited notably low absolute recoveries, primarily attributed to the absence of an appropriate internal standard to account for the extreme matrix effects.

Urinary concentration levels suggest that the frequency of detection of BPA is decreasing while its most prominent alternatives, BPF and BPS, is increasing. Nevertheless, BPA exposure is still found at levels above the TDI, posing a risk to human health. Although no TDI exists for BPF and BPS, they may also represent a relevant human risk. Therefore, actions need to be taken to minimize exposure to these chemicals. Stratification by gender showed significantly higher levels of BPF in women than men.

### Supplementary Information

Below is the link to the electronic supplementary material.

Supplementary file1 (PDF 913 KB)

## Declarations

### Source of biological material

Urine samples were collected from volunteers and anonymized; informed consent from each participant was obtained and stored properly following the requirements established by the ethical approval protocol.
