# Supplementary material for: Determination of the urinary concentrations of six bisphenols in public servants by online solid-phase extraction-liquid chromatography tandem mass spectrometry
Source: Anal Bioanal Chem. 2024 Jun 18;416(20):4469–80. doi: 10.1007/s00216-024-05386-7 (PMC11294393; doi:10.1007/s00216-024-05386-7)
Supplement: Supplementary file 1 — Supplementary file1 (PDF 913 KB) [file 216_2024_5386_MOESM1_ESM.pdf]

Supplementary information

Determination of the urinary concentrations of six bisphenols in public servants by online solid-phase extraction-liquid chromatography-tandem mass spectrometry

Andrea Estévez-Danta*, Rosario Rodil, José Benito Quintana, Rosa Montes*

Aquatic One Health Research Center (ARCUS) & Department of Analytical Chemistry, Nutrition and Food Chemistry. R. Constantino Candeira S/N, IIAA building, Universidade de Santiago de Compostela, 15782 Santiago de Compostela, Spain

For Peer Review

Text S1. Enzymatic hydrolysis optimization	P.3
Text S2. Analytical methodology for creatinine determination in urine.	P.4
Table S1. Chemical structure of target bisphenols	P.5
Table S2. Summary of sociodemographic characteristics and creatinine levels for the studied population.	P.6
Table S3. Linearity parameters, intercept and slope estimates and standard error	P.7
Table S4. Comparison of the performance of the method proposed in this work with other online SPE-LC-MS/MS methods published in the literature.	P. 8-9
Table S5. Comparison of the urinary concentrations with other recent published studies.	P.10-11
Fig. S1 Standardized pareto charts for (a) BPA, (b) BPS and (c) BPF, obtained during enzymatic deconjugation DOE optimization.	P.12
Fig. S2 Estimated response surface for (a) BPF and (b) BPS, obtained during enzymatic deconjugation DOE optimization.	P.13
Fig. S3 Relative response in urine for the target compounds employing different concentrations of NH_4F, as modifier for both LC mobile phases (n=2).	P.14
Fig. S4 Chromatogram of a 100 ng mL^{-1} spiked urine sample under final conditions.	P.15
Fig. S5 Effect of online SPE aqueous phase modifiers (B1) in the peak shape and signal intensity for BPS in urine	P.16
Fig. S6 Stability of bisphenol in urine stored at room temperature. RSD (n=3) < 10%	P.17
Fig. S7 Comparison of creatinine corrected concentrations (µg g^{-1}) according to tobacco use: (a) BPF and (b) BPS	P.18
Fig. S8 Comparison of creatinine corrected concentrations (µg g^{-1}) according to the residence environment: (a) BPF and (b) BPS	P.19

For Peer Review

Text S1. Enzymatic hydrolysis optimization

Design of experiments (DOE)

A central composite design (2^2 + star) with 4 center points was created to make an efficient optimization of the enzyme concentration (experimental domain: 250- 850 units) and incubation time (experimental domain: 1.5 – 4.5 h) variables. The obtained experiments were:

Experiment 1. Enzyme concentration = 550 units, incubation time = 0.88 h

Experiment 2. Enzyme concentration = 250 units, incubation time = 1.5 h

Experiment 3. Enzyme concentration = 850 units, incubation time = 1.5 h

Experiment 4. Enzyme concentration = 126 units, incubation time = 3 h

Experiment 5. Enzyme concentration = 550 units, incubation time = 3 h

Experiment 6. Enzyme concentration = 550 units, incubation time = 3 h

Experiment 7. Enzyme concentration = 550 units, incubation time = 3 h

Experiment 8. Enzyme concentration = 550 units, incubation time = 3 h

Experiment 9. Enzyme concentration = 974 units, incubation time = 3 h

Experiment 10. Enzyme concentration = 250 units, incubation time = 4.5 h

Experiment 11. Enzyme concentration = 850 units, incubation time = 4.5 h

Experiment 12. Enzyme concentration = 550 units, incubation time = 5.12 h

Sample preparation and injection

375 µL of filtered urine was adjusted at pH 5 with sodium acetate buffer and spiked with 100 ng mL^{-1} of available sulfate and glucuronide metabolites mixture (BPA-S, BPA-DS, BPS-S, BPF-S, BPA-G, BPA-DG, BPS-G and BPF-G) + 20 ng mL^{-1} of ISs BPA-d6 and BPS-d8. Then, each experiment was randomly performed according to the conditions specified in the DOE table and injected into the online SPE-LC-MS/MS system following the optimized protocol.

For Peer Review

Text S2. Analytical methodology for creatinine determination in urine.

Sample preparation

Urine samples were filtered through 0.45 µm PVDF syringe-driven filters. Then, each aliquot was diluted 10,000 times in ultrapure water, spiked with the internal standard (creatinine-d3) at 10 ng mL^{-1} and transferred to an insert for injection in the LC-MS/MS system.

Liquid chromatography-tandem mass spectrometry parameters

Instrumental analyses were performed with a Waters Acquity UPLC® H class system (Milford, MA, USA) equipped with a quaternary solvent pump, a thermostated LC column compartment, and a sample manager. The UPLC® system was interfaced to a triple quadrupole mass spectrometer Xevo TQD from Waters.

The chromatographic separation was performed at 30 °C on a Luna C18 column (50 x 2.0 mm, I.D., 3 µm particle size) from Phenomenex. A dual eluent system consisting of (A) 0.1% formic acid in ultrapure water and (B) 0.1% formic acid in MeOH was used at a flow rate of 0.3 mL min^{-1} in isocratic mode (50:50) for 3 minutes. Injection volume was set at 3 µL.

The interface between the UPLC® system and the Xevo TQD mass spectrometer was an electrospray ionization (ESI) source operating in positive mode at a fixed capillary voltage of 3 kV and a temperature of 150 ºC. Nitrogen, provided by a nitrogen generator from Peak Scientific Spain (Barcelona, Spain), was used as desolvation gas at 600 L h^{-1} and 450 °C (desolvation temperature), and as cone gas at 10 L h^{-1}. Analyses were performed by MS/MS in Selected Reaction Monitoring (SRM) mode, where: creatinine SRM transitions 114 > 44 (quantification), 114 > 86 (qualification) and creatinine-d3 SRM transitions 117 > 47 (quantification), 117 > 89 (qualification).

Validation

Calibration curves were prepared in ultrapure water and ranged from the method quantification limit (MQL) to 250 ng mL^{-1}, with IS level of 10 ng mL^{-1}, being the MQL 0.02 ng mL^{-1} and the obtained R^2 was 0.9995. Method repeatability was evaluated at 1 and 50 ng mL^{-1} and the RSD were 7 and 5 %, respectively for 5 consecutive injections. Method accuracy was evaluated through spiking 6 different real urine samples at 2 mg mL^{-1} (which corresponds to 200 mg dL^{-1} in sample and 200 ng mL^{-1} in the diluted urine) and the obtained values were 102 ± 6 %.

For Peer Review

Table S1. Chemical structure of target bisphenols

Name	Structure
**Bisphenol A (BPA)**	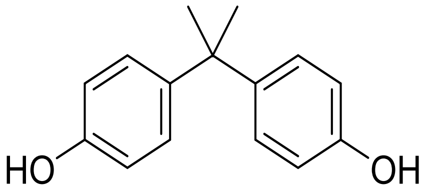
**Bisphenol AF (BPAF)**	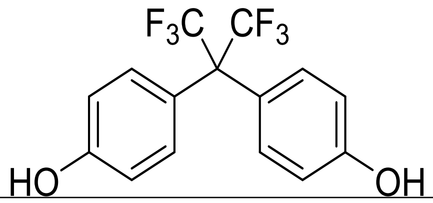
**Bisphenol B (BPB)**	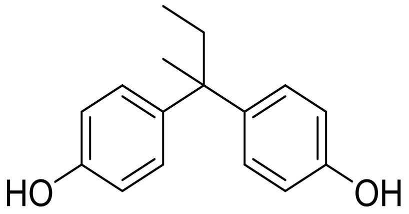
**Bisphenol E (BPE)**	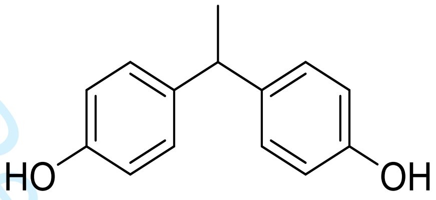
**Bisphenol F (BPF)**	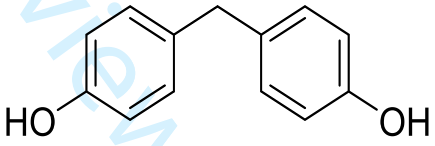
**Bisphenol S (BPS)**	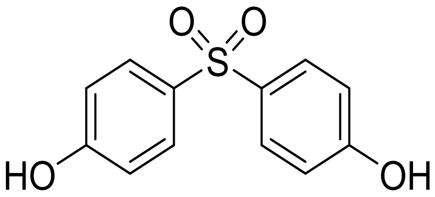

For Peer Review

Table S2. Summary of sociodemographic characteristics and creatinine levels for the studied population (full data presented in ZENODO repository (https://doi.org/10.5281/zenodo.10477935)

Sociodemographic characteristics										Creatinine (g L^{-1})		
Gender %		Tobacco use %		Residence location %			Age					
Female	Male	Yes	No	Urban	Suburban	Rural	Mean	Median	SD	Mean	Median	SD
67.6	32.4	16.1	83.9	56.1	33.8	10.1	51.3	52	7.08	0.94	0.82	0.64

For Peer Review

Table S3. Linearity parameters, intercept and slope estimates and standard error.

	Intercept estimate	Intercept standard error	Slope estimate	Slope standard error	Stardard error of estimate
**BPS**	3.74E-04	7.87E-04	3.80E-03	7.83E-05	3.03E-03
**BPF**	1.43E-03	1.27E-03	7.82E-03	1.31E-04	5.19E-03
**BPE**	-8.49E-04	1.24E-03	1.38E-02	1.30E-04	5.20E-03
**BPA**	2.85E-03	6.08E-04	4.42E-03	6.42E-05	2.56E-03
**BPAF**	-1.94E-03	9.84E-02	3.94E-01	1.04E-02	4.10E-01
**BPB**	-3.84E-03	1.87E-03	2.07E-02	1.98E-04	7.87E-03

Calculations performed using 3 independent calibration curves with Statgraphics Centurion 19 software

For Peer Review

Table S4. Comparison of the performance of the method proposed in this work with other online SPE-LC-MS/MS methods published in the literature.

Reference	Target Bisphenol	Sample preparation	Separation and detection		%R	MQL (ng mL^{-1})
		Pre-treatment	Extraction	LC-MS/MS		
This study	BPA, BPAF, BPB, BPE, BPF and BPS	200 µL of urine<br>Filtration through 0.45 µm PVDF syringe-driven filters<br>Addition of IS + 1mM sodium acetate buffer at pH 5 + 700 units of ß-glucuronidase + 392 µL of ultrapure water<br>Incubation for 5 h at 37 °C	Online SPE on Strata-X 25 µm cartridges<br>Mobile phases: 15 mM of sodium acetate buffer at pH 5 in ultrapure water- MeOH<br>Inj. Vol.: 500 µL	LC-(ESI-)-MS/MS on QqQ (SRM)<br>Luna C18 (150 x 2 mm I.D., 3 µm)<br>Mobile phase: 2 mM of NH_4F in ultrapure water – 2 mM of NH_4F in MeOH	92-112 % (except BPAF 11-40%)	0.049-2.2
Ye et al., 2005	BPA	100 µL of urine<br>Addition of IS + 50 µL of enzyme/ ammonium acetate Incubation overnight at 37 °C	Online SPE on LiChrosphere RP-18 ADS 25 µm column<br>Mobile phases: ultrapure water- MeOH<br>Inj. Vol.: 1000 µL	LC-(APCI-)-MS/MS on QqQ (MRM)<br>Chromolith Performance RP-18 (100 x 4.6 mm I.D.)<br>Mobile phases: ultrapure water- MeOH	100 %	1.3
Koch et al., 2012	BPA	300 µL of urine<br>Addition of IS + 1 mM ammonium acetate buffer at pH 5 + 6 µL of ß-glucuronidase<br>Incubation for 4 h at 37 °C	Online SPE on LiChrosphere RP-8 ADS 25 µm column<br>Mobile phases: ultrapure water- Acetonitrile<br>Inj. Vol.: 100 µL	HPLC-(ESI-)-MS/MS on QTRAP (MRM)<br>Waters Atlantis T3 analytical column (150 x 3 mm I.D., 3 µm)<br>Mobile phases: ultrapure water-Acetonitrile	96.8%	0.1
Zhou et. al., 2014	BPA, BPF and BPS	100 µL of urine<br>Addition of IS + dilution to 1 mL with 0.1 M formic acid + 50 µL of ß-glucuronidase/sulfatase<br>Incubation for 4 h at 37 °C<br>Stop solution: 750 µL 0.1 M formic acid in ultrapure water	Online SPE on LiChrosphere RP-18 ADS 25 µm column<br>Mobile phases: ultrapure water- MeOH<br>Inj. Vol.: 350 µL	LC-(APCI-)-MS/MS on QqQ (MRM)<br>Chromolith High Resolution RP-18e (100 x 4.6 mm I.D.)<br>Mobile phases: ultrapure water- MeOH	77-106%	0.03-0.1

For Peer Review

Heffernan et al., 2016	BPA, BPAF, BPB, BPF and BPS	50 µL of urine<br>Addition of IS + 25 µL of ß-glucuronidase (200 units) + 440 µL ultrapure water<br>Incubation for 90 min at 37 °C<br>Stop solution: 400 µL of 0.5 % formic acid in ultrapure water.	Online SPE on Strata-X 25 µm cartridges<br>Mobile phase: 0.05 % of acetic acid in ultrapure water: 0.05 % of acetic acid in MeOH<br>Inj. Vol.: 500 µL	LC-(ESI-)-MS/MS on QTRAP (MRM)<br>Synergi MAX-RP column (150 x 3 mm I.D., 4 µm)<br>Mobile phase: 0.05% of acetic acid in ultrapure water: 0.05 % of acetic acid in MeOH	101-110%	0.005-0.39
Jo et al., 2020	BPA, BPF and BPS	100 µL of urine<br>Addition of IS + 100 µL of ß-glucuronidase/sulfatase (1000 units)<br>Incubation for 24h at 37 °C<br>Stop solution: 80 µL 1 M formic acid + 670 µL ultrapure water	Online SPE on MAYI-ODS column 50 µm<br>Mobile phases: ultrapure water- MeOH<br>Inj. Vol.: 100 µL	LC-(APCI-)-MS/MS on QqQ (MRM)<br>ACE 5 C18-pentafluorophenyl column (150 x 2.1 mm I.D., 5 µm)<br>Mobile phases: ultrapure water- MeOH	99.4-108%	0.13-0.24

References:
1. Ye X, Kuklenyik Z, Needham LL, Calafat AM. Automated on-line column-switching HPLC-MS/MS method with peak focusing for the determination of nine environmental phenols in urine. Analytical Chemistry 2005; 77: 5407-5413.
2. Koch HM, Kolossa-Gehring M, Schröter-Kermani C, Angerer J, Brüning T. Bisphenol A in 24 h urine and plasma samples of the German Environmental Specimen Bank from 1995 to 2009: a retrospective exposure evaluation. J Expo Sci Environ Epidemiol 2012; 22: 610-6.
3. Zhou X, Kramer JP, Calafat AM, Ye X. Automated on-line column-switching high performance liquid chromatography isotope dilution tandem mass spectrometry method for the quantification of bisphenol A, bisphenol F, bisphenol S, and 11 other phenols in urine. Journal of Chromatography B 2014; 944: 152-156.
4. Heffernan AL, Thompson K, Eaglesham G, Vijayasarathy S, Mueller JF, Sly PD, et al. Rapid, automated online SPE-LC-QTRAP-MS/MS method for the simultaneous analysis of 14 phthalate metabolites and 5 bisphenol analogues in human urine. Talanta 2016; 151: 224-233.
5. Jo MJ, Park J-H, An K-A, Choi H, Kang Y-s, Hwang M. Quantification of bisphenols in Korean urine using online solid-phase extraction-high-performance liquid chromatography-tandem mass spectrometry. Environmental Toxicology and Pharmacology 2020; 80: 103491.

For Peer Review

Table S5. Comparison of the urinary concentrations with other recent published studies.

Reference	Study period	City	Sub-population type	Target Bisphenol	N	Positive samples	Geometric mean (GSD) concentration ng mL^{-1} [a]	Min-Max ng mL^{-1}	Geometric mean (GSD) adjusted concentration [a]
This study	Sep 2020	Santiago de Compostela (Spain)	General population (67.6 % women / 36.4% men)	BPA, BPAF, BPB, BPE, BPF and BPS	435	**BPA**: 72<br>**BPS**: 364<br>**BPF:** 421<br>**BPAF, BPB, BPE:** 0	**BPA**: -<br>**BPS**: 0.50 (8.66)<br>**BPF:** 12.4 (3.1)	**BPA**: MQL-103<br>**BPS**: MQL-80<br>**BPF:** MQL-125	**Creatinine-correction (µg g^{-1}):**<br>**BPA:** -<br>**BPS:** 0.67 (6.64)<br>**BPF:** 16.6 (2.6)
Peinado et al., 2020	Jan 2018-Jul 2019	Granada (Spain)	Women population	BPA, BPS and BPF	35	**BPA**: 35<br>**BPS**: 4<br>**BPF:** 10	**BPA**: 5.5 (1.1)<br>**BPS**: 0.1 (1.1)<br>**BPF**: 0.1 (1.2)	**BPA**: 0.8-18<br>**BPS**: 0.1-1.5<br>**BPF:** 0.1-0.9	**Creatinine-correction (µg g^{-1}) [b]:**<br>**BPA**: 4.7 (1.1)<br>**BPS**: 0.086 (1.100)<br>**BPF**: 0.086 (1.200)
Sanchis et al., 2020	Jun-Nov 2015	Valencia (Spain)	Breastfeeding mothers	BPA, BPS and BPF	103	**BPA**: 78<br>**BPS**: 21<br>**BPF:** 21	**BPA**: 0.9 (3)<br>**BPS**: 0.06 (0.85)<br>**BPF**: 0.04 (0.34)	**BPA**: LOQ-3<br>**BPS**: LOQ-8.5<br>**BPF:** LOQ-0.34	**Creatinine-correction (µg g^{-1}):**<br>**BPA**: 2.7 (4.7)<br>**BPS**: 0.29 (1.40)<br>**BPF**: 0.19 (0.37)
Heffernan et al., 2016	2015	Brisbane (Australia)	Pregnant women	BPA, BPAF, BPB, BPF and BPS	30	**BPA**: 30<br>**BPS**: 3<br>**BPF:** 3<br>**BPAF, BPB:** 0	**BPA**: 5.0<br>**BPS: -**<br>**BPF**: -	**BPA**: 1.7-45<br>**BPS**: MQL-8.1<br>**BPF:** MQL-74	-
Husøy et al., 2019	Sep 2016-Nov 2019	Oslo and Akershus (Norway)	General population (69.4 % women / 30.6% men)	BPA, BPS and BPF	144	**BPA**: 138<br>**BPS**: 42<br>**BPF:** 6	-	**BPA**: 0.2-10<br>**BPS**: 0.04-13<br>**BPF:** MDL-9.9	**Specific gravity-correction (ng mL^{-1}):**<br>**BPA**: 1.4<br>**BPS**: 0.2<br>**BPF**: 0.1
Frederisken et al., 2020	2009	Copenhagen (Denmark)	Men population	BPA, BPS, BPF	100	**BPA**: 100<br>**BPS**: 68<br>**BPF:** 81	-	**BPA**: 2.2-24<br>**BPS**: 0.09-5.2<br>**BPF:** 0.27-4.5	**Osmolality-correction (ng mL^{-1}):**<br>**BPA**: 2.27<br>**BPS**: 0.11<br>**BPF:** 0.30

For Peer Review

2013	Men population	100	**BPA**: 90<br>**BPS**: 65<br>**BPF**: 78	-	**Osmolality-correction ng mL^{-1}:**<br>**BPA**: 1.44<br>**BPS**: 0.06<br>**BPF:** 0.24
2017	Men population	100	**BPA**: 92<br>**BPS**: 86<br>**BPF**: 87	-	**Osmolality-correction ng mL^{-1}:**<br>**BPA**: 1.33<br>**BPS**: 0.18<br>**BPF:** 0.32

[a] Geometric mean and geometric standard deviation were calculated by substituting MQL/2 or MDL/2 for values <MDL/MQL.

[b] Calculated from the data reported in the article

References:

1. Peinado FM, Lendínez I, Sotelo R, Iribarne-Durán LM, Fernández-Parra J, Vela-Soria F, et al. Association of Urinary Levels of Bisphenols A, F, and S with Endometriosis Risk: Preliminary Results of the EndEA Study. International Journal of Environmental Research and Public Health 2020; 17: 1194.
2. Sanchis Y, Coscollà C, Corpas-Burgos F, Vento M, Gormaz M, Yusà V. Biomonitoring of bisphenols A, F, S and parabens in urine of breastfeeding mothers: Exposure and risk assessment. Environmental Research 2020; 185: 109481.
3. Heffernan AL, Thompson K, Eaglesham G, Vijayasarathy S, Mueller JF, Sly PD, et al. Rapid, automated online SPE-LC-QTRAP-MS/MS method for the simultaneous analysis of 14 phthalate metabolites and 5 bisphenol analogues in human urine. Talanta. 2016; 151:224-33
4. Husøy T, Andreassen M, Hjertholm H, Carlsen MH, Norberg N, Sprong C, et al. The Norwegian biomonitoring study from the EU project EuroMix: Levels of phenols and phthalates in 24-hour urine samples and exposure sources from food and personal care products. Environment International. 2019;132.
5. Frederiksen H, Nielsen O, Koch HM, Skakkebaek NE, Juul A, Jørgensen N, et al. Changes in urinary excretion of phthalates, phthalate substitutes, bisphenols and other polychlorinated and phenolic substances in young Danish men; 2009–2017. International Journal of Hygiene and Environmental Health. 2020;223(1):93-105.

For Peer Review

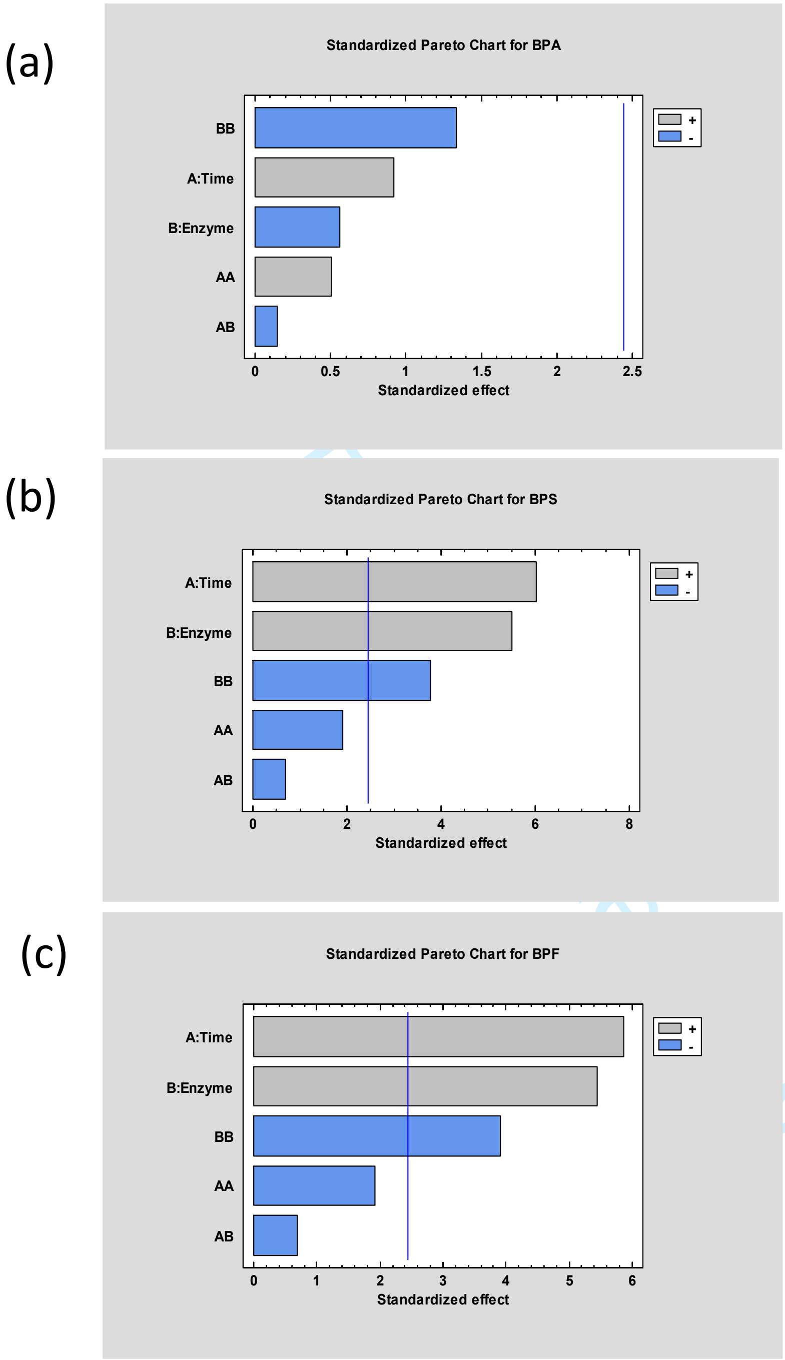

Fig. S1 Standardized pareto charts for (a) BPA, (b) BPS and (c) BPF, obtained during enzymatic deconjugation DOE optimization

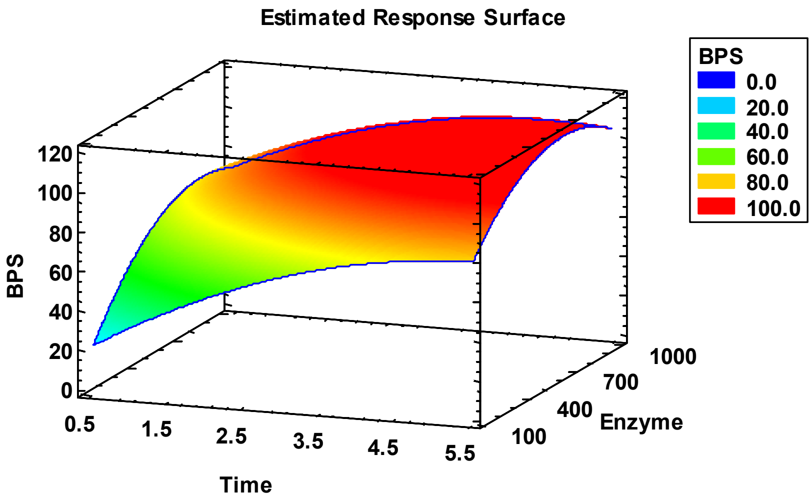

Fig. S2 Estimated response surface for BPS, obtained during enzymatic deconjugation DOE optimization

eer Review

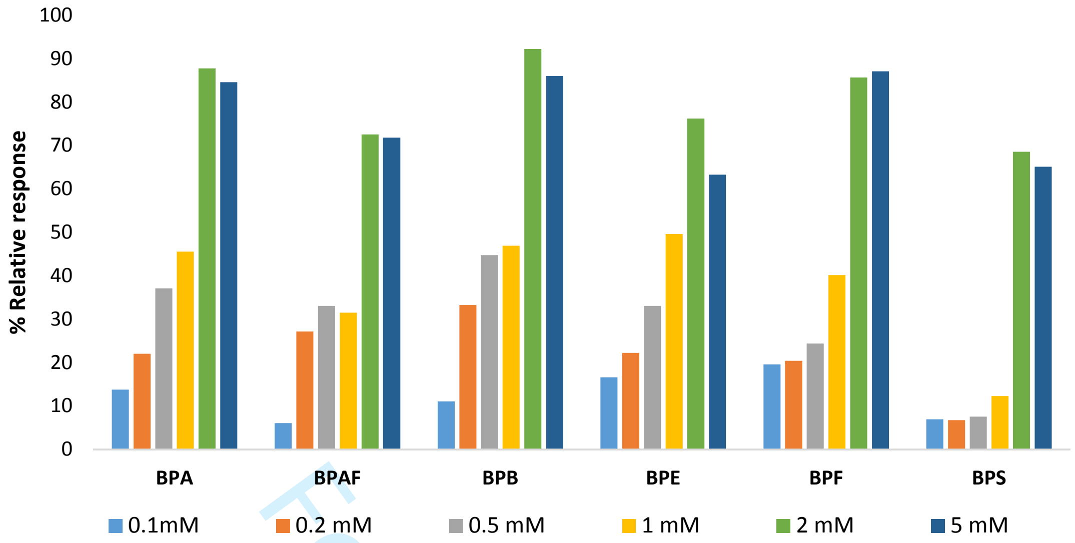

Fig. S3 Relative response in urine for the target compounds employing different concentrations of NH_4F, as modifier for both LC mobile phases (n=2).

For Peer Review

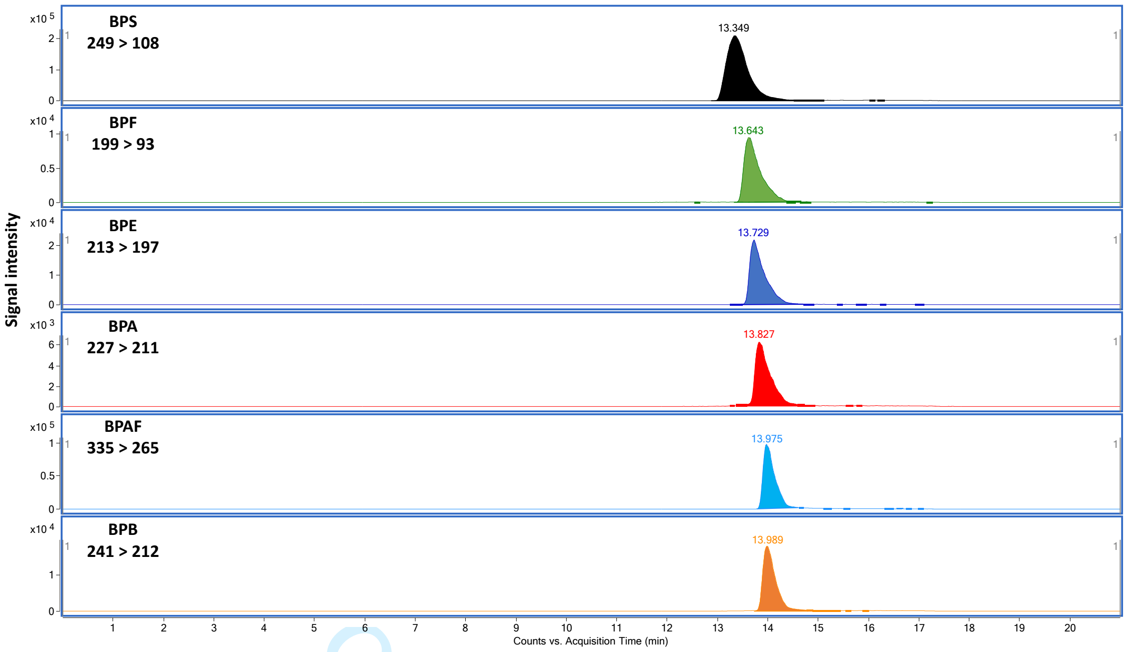

Fig. S4 Chromatogram of a 100 ng mL^{-1} spiked urine sample under final conditions.

For Peer Review

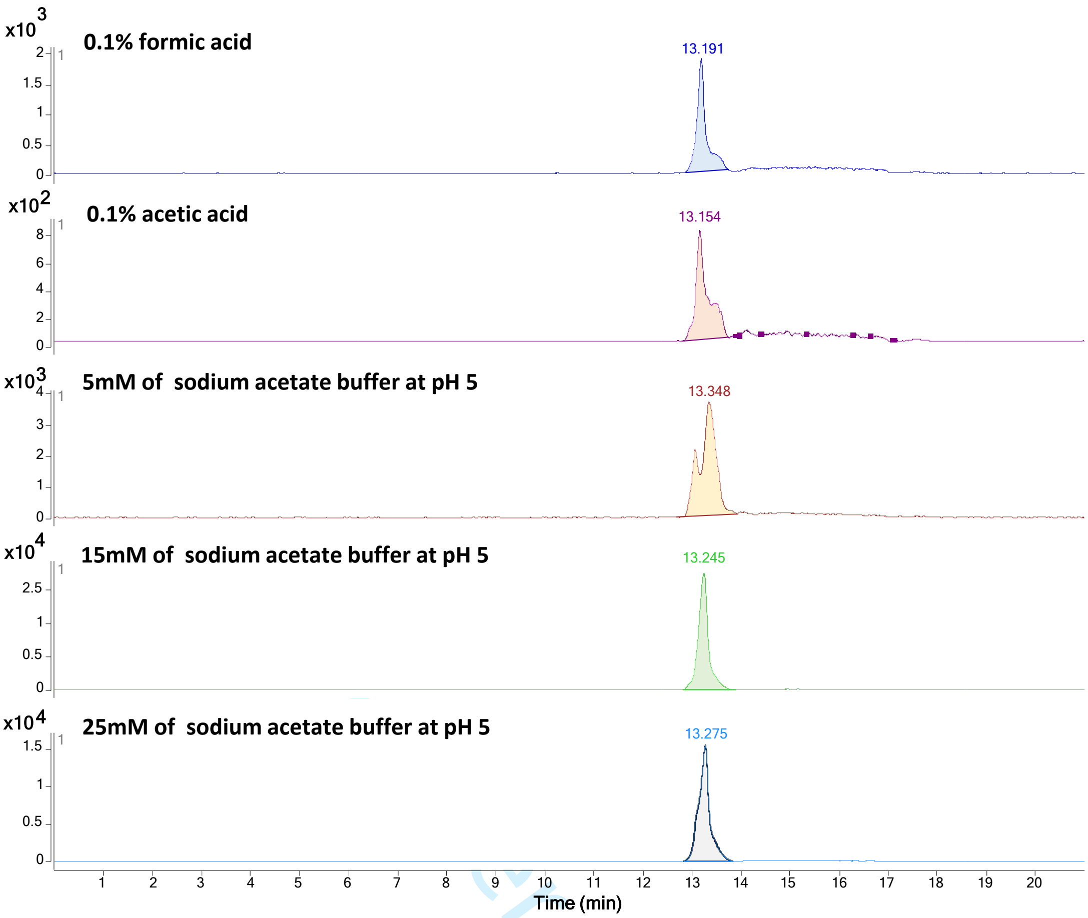

Fig. S5 Effect of online SPE aqueous phase modifiers (B1) in the peak shape and signal intensity for BPS in urine

er Review

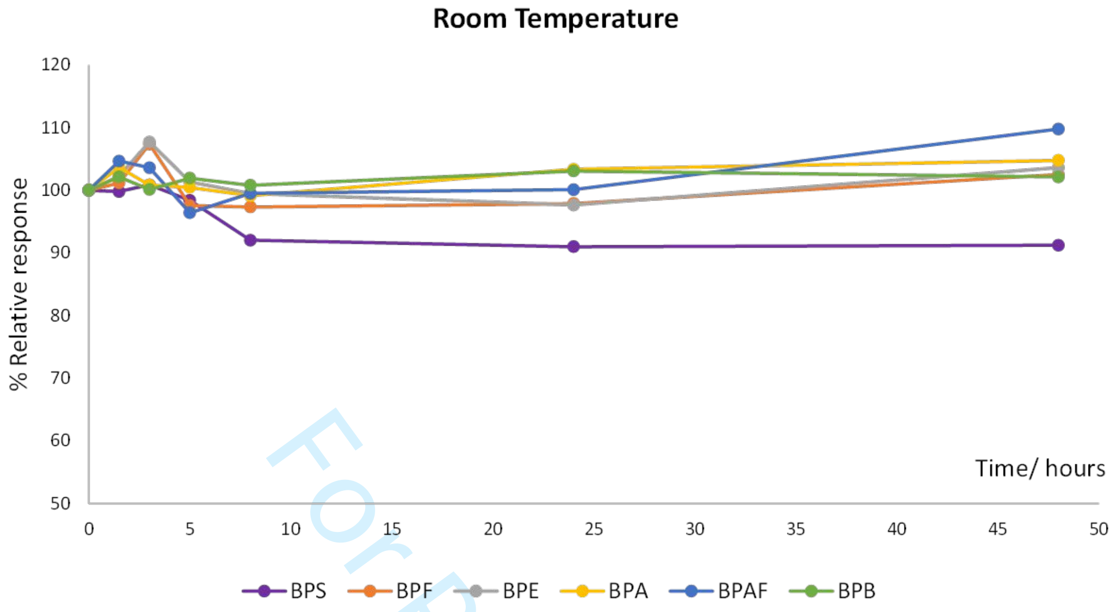

Fig. S6 Stability of bisphenol in urine stored at room temperature. RSD (n=3) < 10%

For Peer Review

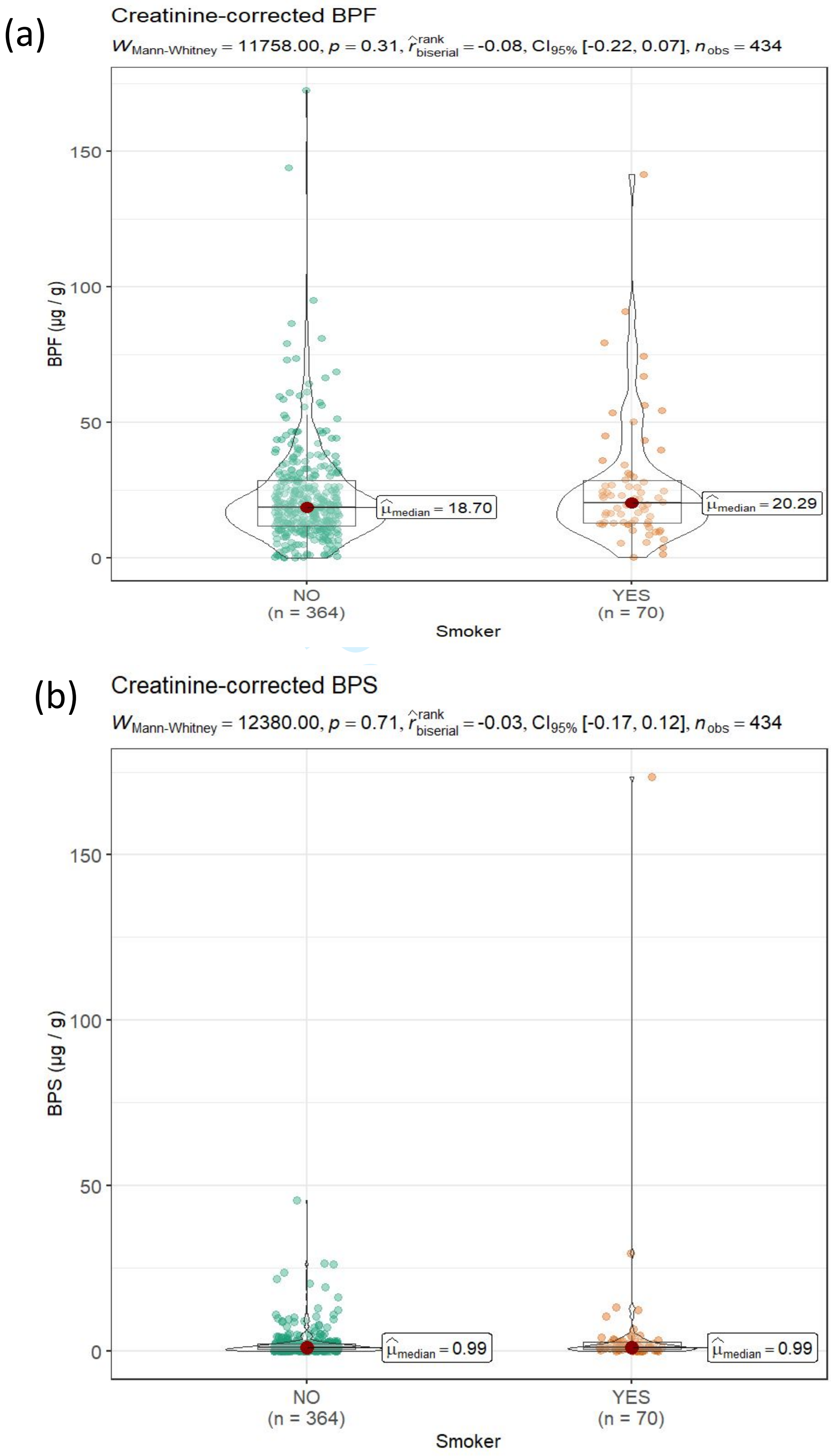

Fig. S7 Comparison of creatinine corrected concentrations (µg g^{-1}) according to tobacco use: (a) BPF and (b) BPS

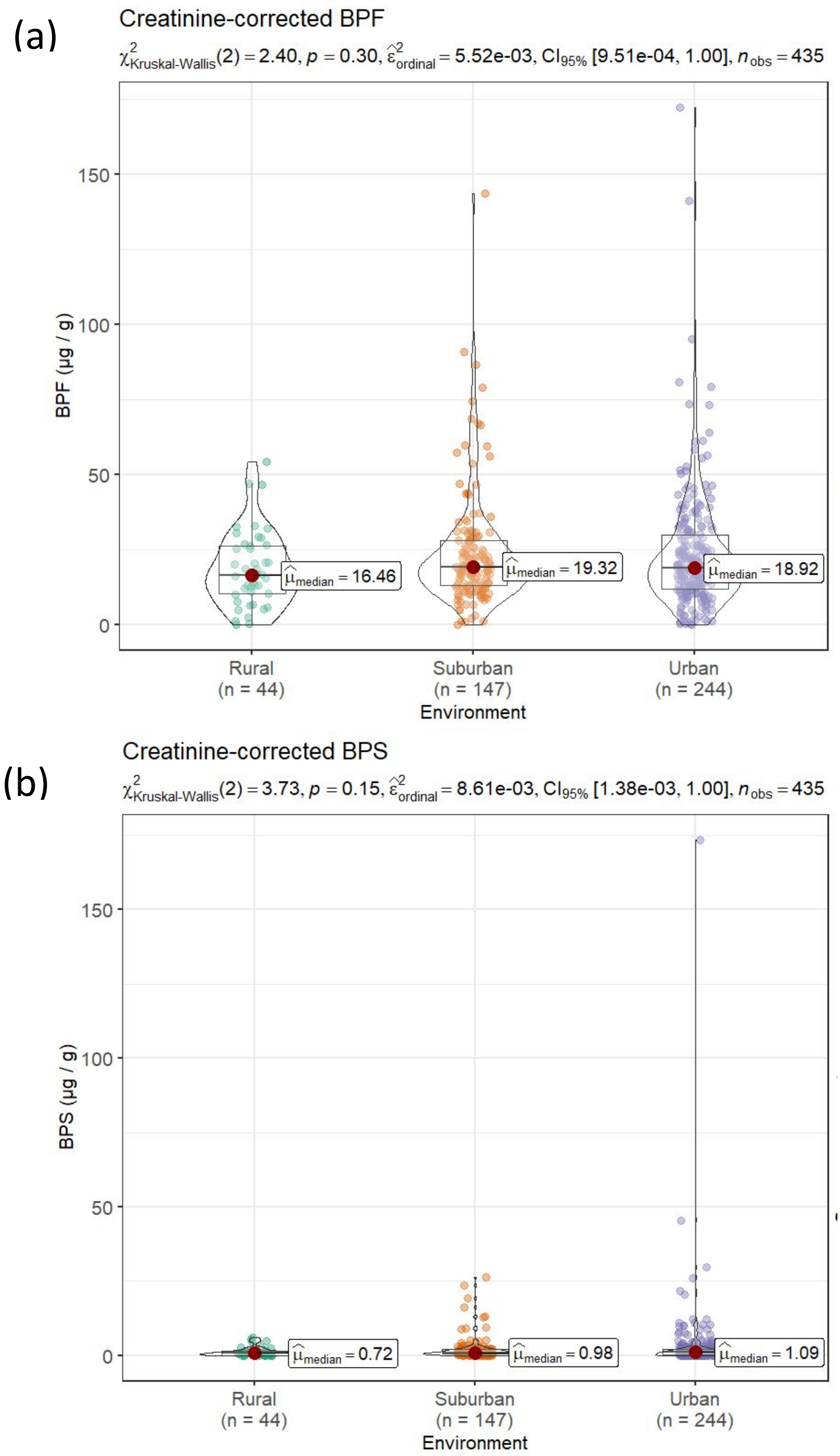

Fig. S8 Comparison of creatinine corrected concentrations (μg g^{-1}) according to the residence environment: (a) BPF and (b) BPS
